# The prognostic role of Sirt1 expression in solid malignancies: a meta-analysis

**DOI:** 10.18632/oncotarget.18494

**Published:** 2017-06-15

**Authors:** Changwen Wang, Wen Yang, Fang Dong, Yawen Guo, Jie Tan, Shengnan Ruan, Tao Huang

**Affiliations:** ^1^ Department of Breast and Thyroid Surgery, Union Hospital, Tongji Medical College, Huazhong University of Science and Technology, Wuhan, China

**Keywords:** Sirt1, solid malignancy, prognosis, meta-analysis

## Abstract

Although many studies have discussed the association of abnormally expressed silent information regulator 1 (Sirt1) with the prognosis of patients with a variety of solid carcinomas, they failed to agree on whether excessive Sirt1 indicates a good or poor overall survival for the patients. We conducted the current meta-analysis to illustrate the prognostic value of Sirt1 in solid malignancies. Articles published before December 2016 were searched using Pubmed and Web of Science. The studies were selected for the meta-analysis based on certain criteria. A total of 7,369 cases from 37 studies were included, in which 48.6% of the patients overexpressed Sirt1. The overall survival (OS) and clinical features, such as age and TNM stage, were analyzed using RevMan 5.3 software. Sirt1 overexpression was significantly correlated with the OS (HR: 1.52, 95% CI: [1.23, 1.88], *P* = 0.0001), especially in liver cancer (HR: 1.78, 95% CI: [1.46, 2.18], *P* < 0.00001) and lung cancer (HR: 1.80, 95% CI: [1.06, 3.05], *P* = 0.03), which suggested that the overexpression of Sirt1 indicates poor prognosis of patients with solid cancers.

## INTRODUCTION

The increase in cancer prevalence and mortality and its impact on social economy have drawn enormous attention towards investigations into the occurrence, development and metastasis of cancer [1]. A large number of cell signaling pathways have been discovered and studied, and accumulating evidence has shown that epigenetic regulation of gene expression contributes significantly to solid malignancy [2]. Aberrant activation of key epigenetic pathways, including Sirt1 signaling, contributes to carcinogenesis in a variety of tumors, suggesting a potential therapeutic target for future treatments [3].

Sirt1, a proto member of the sirtuin family, is an NAD^+^-dependent histone deacetylase. Sirt1 modifies histones and non-histone proteins through deacetylation [4]. Sirt1 plays pivotal roles in a variety of physiological processes, such as cell metabolism, proliferation, senescence, apoptosis, and tumorigenesis [3, 4]. It exercises its functions through p53 [5], FoxO1 [6], NF-*κ*B [7] and other signaling pathways. Sirt1, because of its tumorigenic characteristics, can be targeted for therapy, which may provide a longer lifespan and better quality of life for cancer patients. Interestingly, Sirt1 seems to have dual roles in cancer. Sirt1 promotes tumorigenesis by boosting cell survival under stress conditions but facilitates uncontrolled cell proliferation, and additionally, it can defend against carcinomas by increasing genomic stability and limiting cellular replicative lifespan [8]. Additionally, Sirt1 expression is increased in ovarian cancer [9] and gastric cancer [10], whereas it is reduced in liver cancer and breast cancer [11]. Therefore, it remains controversial whether Sirt1 overexpression indicates a good or poor prognosis. Although the majority of the evidence shows that overexpressed Sirt1 has a negative prognostic effect on cancer patients, Jung et al. [12] failed to draw the same conclusion, and their data suggested that higher Sirt1 expression resulted in a better survival status. Consequently, the clinical significance of SIRT1 in cancers is complex and requires further investigation. Therefore, in the present study, we conducted an exhaustive meta-analysis and subgroup analyses to understand the prognostic effects of Sirt1 overexpression in solid malignancies, with the aim to provide evidence for improved targeted regimens.

## RESULTS

### Eligible studies

**Figure 1:**
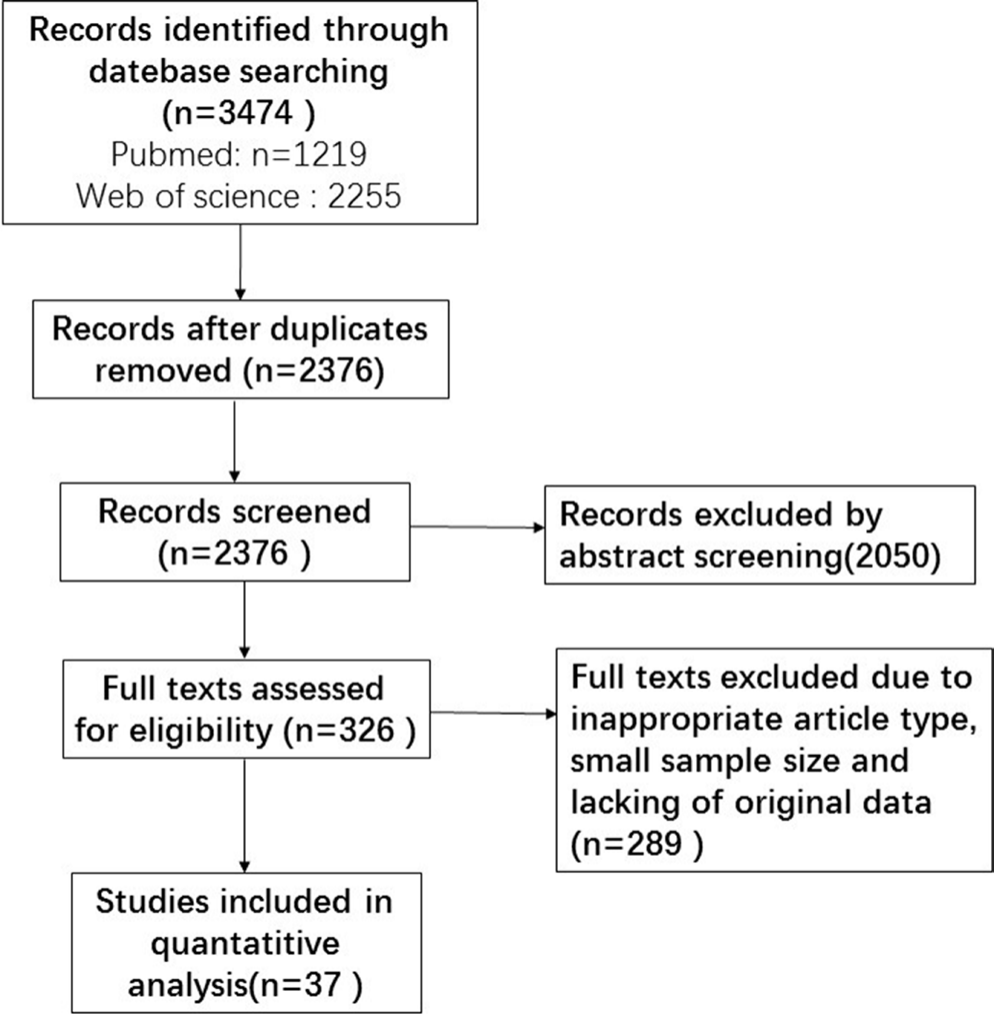
Flow chart of the selection for the meta-analysis

Most of the studies found during the initial search were excluded based on the selection criteria such as inappropriate article type, replicated data or insufficient original information. Eventually, 37 qualified studies containing 7,369 cases were included for analyses. Figure 1 shows the selection workflow of eligible studies for our meta-analysis.

### Demographic characteristics of the included studies

Among the 37 studies, the majority (15) of them were conducted in China, followed by Korea (*n* = 13) and other countries. The majority of the studies were based on breast cancer (*n* = 8), followed by colorectal carcinoma, hepatocellular carcinoma, gastric cancer (*n* = 5, respectively), lung cancer (*n* = 4), and other types of solid carcinoma. The sample sizes ranged from 40 to 557, with a median of 144 patients. Among the 37 studies, 34 studies described the correlation between overall survival and Sirt1 expression, 9 trials demonstrated the relationship between disease-free survival and Sirt1 expression, 6 studies discussed relapse-free survival and Sirt1 expression, and 3 articles studied the correlation of cancer-specific survival and abnormal expression of Sirt1. Other details and features were recorded and are summarized in Table 1. All the eligible entries scored higher than six by NOS, suggesting a high methodological quality across all studies.

**Table 1:** Demographic information of included studies

Reference	Country	Cancer type	No.	Male/Femaleale	TNM Stagee	Sirt1 high (+)	Sirt1 low	Follow-up range months	NOS score
Zhang 2016 [13]	China	breast cancer	149	All female	I-III	68	81	NA	7
Chen 2014 [14]	China	colorectal adenocarcinoma	102	56/46	II-IV	44	58	NA	7
Jang 2012 [15]	South Korea	colorectal adenocarcinoma	497	281/216	I-IV	208	289	NA	8
Jung 2013 [12]	South Korea	colorectal adenocarcinoma	349	208/141	I-IV	235	114	NA	8
Cheng 2016 [16]	China	colorectal adenocarcinoma	90	47/43	I-IV	37	53	NA	7
He 2016 [17]	China	esophageal squamous cell carcinoma	86	64/22	I-III	54	32	NA	7
Chen 2014a [18]	China	esophageal squamous cell carcinoma	206	152/54	NA	95	111	5–86	7
Zhang 2013 [19]	China	gastroesophageal junction cancer	90	NA	NA	46	44	NA	7
Cha 2009 [10]	South Korea	gastric carcinoma	177	135/42	I-IV	130	47	NA	7
Cao 2014 [20]	China	breast cancer	122	All female	I-IV	94	28	2–161	7
Kang 2012 [21]	South Korea	gastric carcinoma	452	309/143	I-IV	255	197	NA	7
Feng 2011 [22]	China	gastric carcinoma	90	NA	NA	46	44	NA	7
Noguchi 2014 [23]	Japan	gastric carcinoma	557	391/166	I-IV	345	212	6–142	8
Hao 2014 [24]	China	hepatocellular carcinoma	99	89/10	I-IV	76	23	NA	6
Song 2014 [25]	China	hepatocellular carcinoma	300	267/33	I-IV	145	155	3–83	7
Jang 2012 [18]	South Korea	hepatocellular carcinoma	154	132/22	I-IV	55	99	NA	7
Li 2016 [26]	China	hepatocellular carcinoma	72	65/7	I-III	41	31	NA	6
Chen 2012 [27]	China	hepatocellular carcinoma	172	142/30	NA	95	77	45–236	7
Noguchi 2013 [28]	Japan	head and neck squamous cell carcinoma	437	356/81	NA	348	89	1–174	8
Chung 2015 [29]	South Korea	breast cancer	427	All female	NA	227	150	NA	7
Yu 2013 [30]	China	laryngeal and hypopharyngeal carcinomas	46	NA	NA	17	29	NA	7
Grbesa 2015 [31]	Spain	lung cancer	105	93/12	NA	52	53	NA	7
Noh 2013 [32]	South Korea	lung cancer	144	82/62	NA	75		40–136	7
Li 2015 [33]	China	lung cancer	75	39/36	I-IV	56	19	NA	7
Shin 2016 [34]	South Korea	ovarian cancer	45	NA	NA	16	29	NA	6
Lee 2010 [35]	South Korea	breast cancer	122	All female	NA	82	40	NA	7
Mvunta 2016 [36]	Japan	ovarian cancer	68	All female	NA	11	57	NA	7
Stenzinger 2013 [37]	Germany	pancreatic cancer	113	NA	NA	32	81	NA	7
Noh 2013 [38]	South Korea	renal cell carcinoma	200	140/60	I-IV	119	81	NA	7
Batra 2016 [39]	India	retinoblastoma	94	62/32	NA	49	45	NA	6
Kim 2013 [40]	South Korea	soft tissue sarcomas	104	59/45	NA	74	30	NA	7
Benard 2015 [41]	Dutch	colorectal adenocarcinoma	254	128/126	I-III	NA	NA	NA	6
Chung 2016 [42]	South Korea	breast cancer	344	All female	NA	146	198	NA	8
Jin 2016 [43]	South Korea	breast cancer	319	All female	I-III	107	212	NA	7
Wu 2012 [44]	China	breast cancer	134	All female	NA	72	62	NA	6
Gharabaghi 2016 [45]	Iran	lung cancer	40	23/17	NA	27	13	NA	6
Derr 2014 [46]	Dutch	breast cancer	460	All female	I-III	NA	NA	2–330	7

NOS: Newcastle-Ottawa Scale.

NA: not available.

### Correlation of Sirt1 expression with the overall survival and subgroup analyses

**Figure 2:**
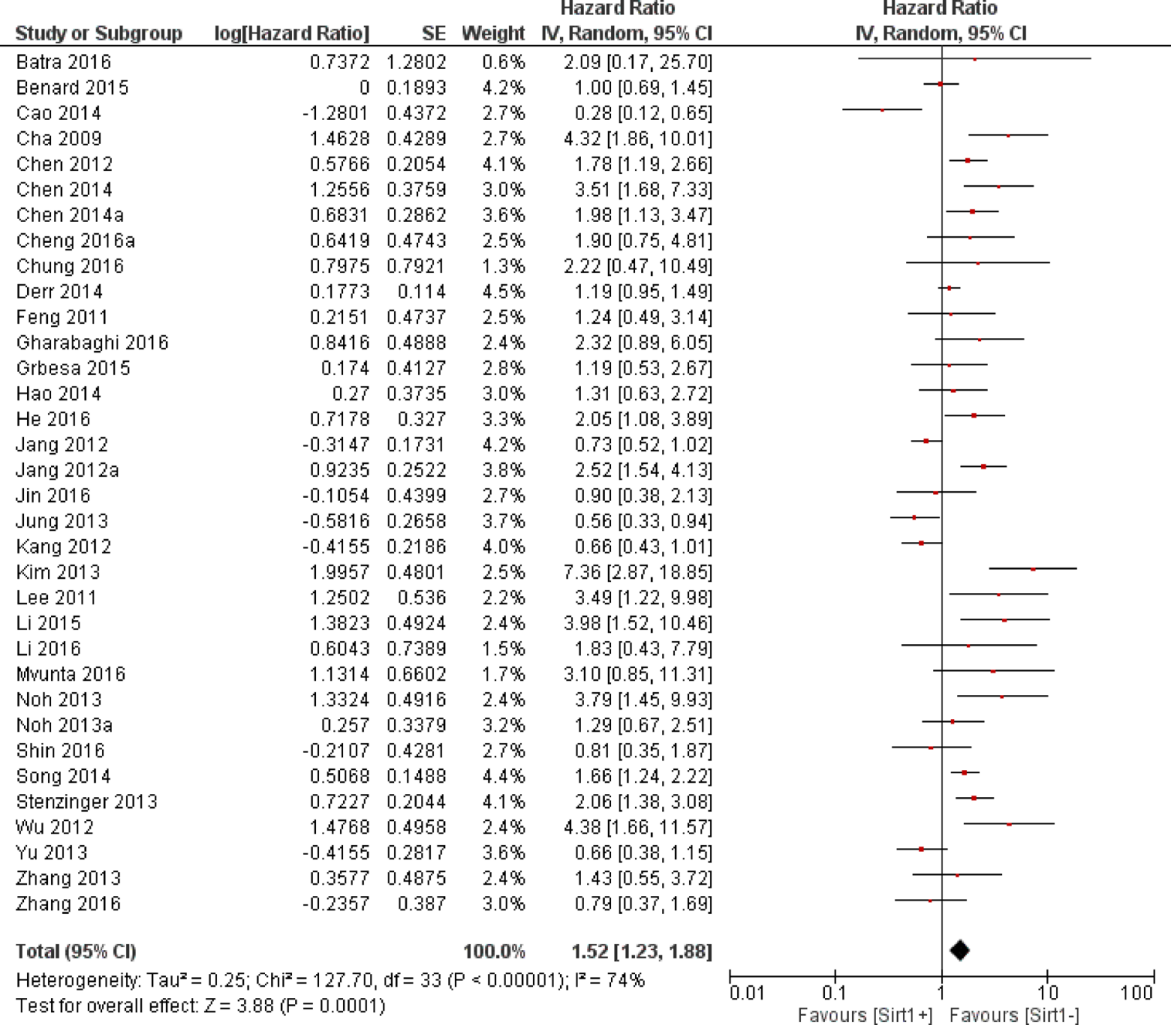
The correlation between Sirt1 expression and overall survival in solid malignancies

**Figure 3:**
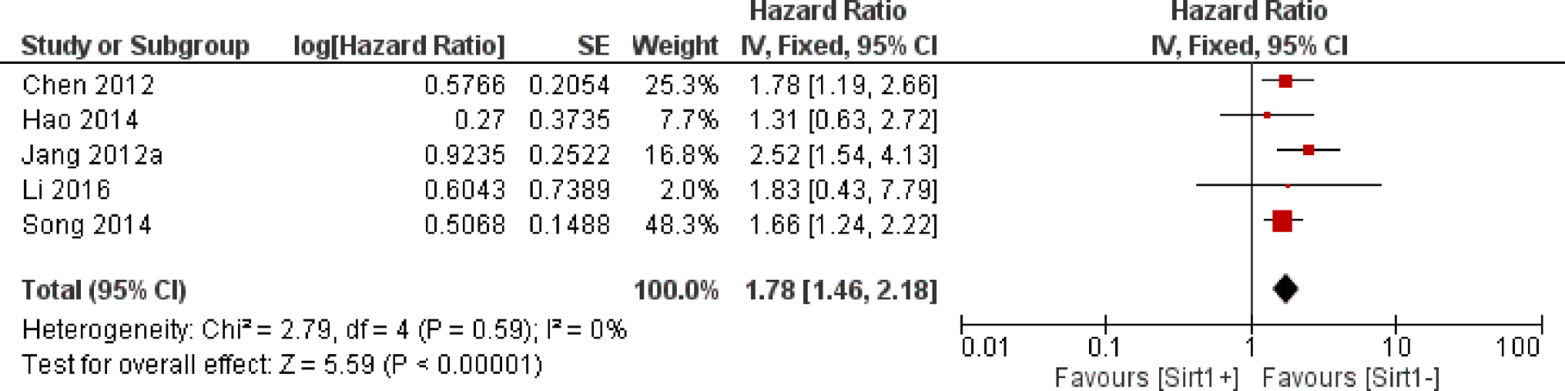
The correlation between Sirt1 expression and overall survival of liver cancer

**Figure 4:**
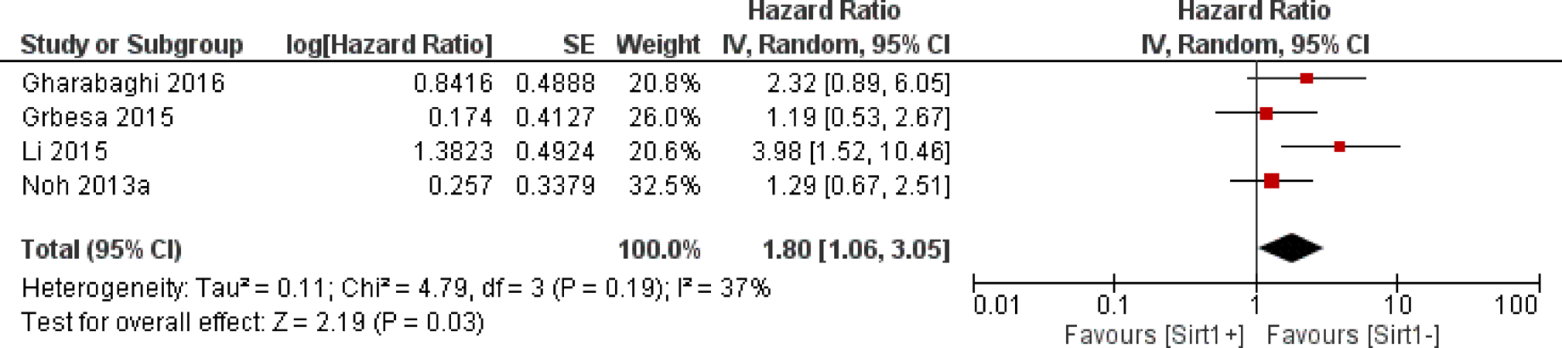
The correlation between Sirt1 expression and overall survival of lung cancer

Thirty-four trials offered data on the correlation between the overall survival and Sirt1 expression. Our calculations showed that higher Sirt1 expression indicated an unfavorable overall survival for solid malignancies (HR: 1.52, 95% CI: [1.23, 1.88], *P* = 0.0001, Figure 2). Because of the significant heterogeneity (*I*^2^ = 74%), we applied a random-effects model for the statistical analysis. To determine possible sources of heterogeneity among studies, we grouped the original articles for subgroup analyses, based on several factors. In the cancer subgroup, Sirt1 overexpression was associated with a worse overall survival in liver cancer (*n* = 5, HR: 1.78, 95% CI: [1.46, 2.18], *P* < 0.00001, *I*^2^ = 0%, Figure 3) and lung cancer (*n* = 4, HR: 1.80, 95% CI: [1.06, 3.05], *P* = 0.03, *I*^2^ = 37%, Figure 4), whereas Sirt1 overexpression was not correlated with the overall survival in breast cancer (*n* = 7, HR: 1.25, 95% CI: [0.70, 2.22], *P* = 0.46, *I*^2^ = 75%, Supplementary Figure 1), colorectal carcinoma (*n* = 5, HR: 1.12, 95% CI: [0.66,1.89], *P* = 0.67, *I*^2^ = 81%, Supplementary Figure 2), and gastric carcinoma (*n* = 4, HR: 1.44, 95% CI: [0.60, 3.45], *P* = 0.41, *I*^2^ = 81%, Supplementary Figure 3). When the studies were sub grouped based on TNM clinical stages, we found several studies that discussed pre-terminal stages (TNM I-III) (*n* = 6, HR: 1.16, 95% CI: [0.98, 1.38], *P* = 0.09, *I*^2^ = 8%, Supplementary Figure 4), and only one study [14] that included advanced TNM stages (II-IV) with HR: 3.51, 95% CI: (1.68, 7.33). Moreover, in studies that covered all stages (TNM I-IV), overexpression of Sirt1 suggested a poorer overall outcome (*n* = 12, HR: 1.35, 95% CI: [0.87, 2.11], *P* = 0.18, *I*^2^ = 86%, Supplementary Figure 5). Two studies evaluated the nuclear and cytoplasmic Sirt1 expressions separately, with distinctive results. One article showed that overexpression of Sirt1 in the cytoplasm is indicative of a good prognosis (*P* < 0.005), whereas another suggested that increased expression of Sirt1 significantly correlated with poor patient survival (HR = 2.617, *P* = 0.019). The remaining studies detected the nuclear expression of Sirt1 expression (*n* = 17, HR: 1.56, 95% CI: [1.15, 2.12], *P* = 0.004, *I*^2^ = 80%).

### Correlation of Sirt1 expression with disease-free survival, relapse-free survival and cancer-specific survival

Unfortunately, our analysis failed to conclude the significance between increased Sirt1 expression and relapse-free survival (RFS, *n* = 6, HR: 1.58, 95% CI: [0.97, 2.60], *P* = 0.07, *I*^2^ = 84%, Supplementary Figure 6), disease-free survival (DFS, *n* = 9, HR: 1.23, 95% CI: [0.88, 1.73], *P* = 0.22, *I*^2^ = 71%, Supplementary Figure 7) or cancer-specific survival (CSS, *n* = 3, HR: 1.40, 95% CI: [0.60, 3.30], *P* = 0.44, *I*^2^ = 89%, Supplementary Figure 8) among solid malignancies.

### Sensitivity analysis

Excluding studies about breast cancer (*n* = 27, HR: 1.60, 95% CI: [1.26, 2.04], *P* = 0.0001, *I*^2^ = 74%), colorectal cancer (*n* = 29, HR: 1.62, 95% CI: [1.29, 2.03], *P* < 0.0001, *I*^2^ = 70%), and gastric cancer (*n* = 30, HR: 1.54, 95% CI: [1.23, 1.92], *P* = 0.0001, *I*^2^ = 74%) had no substantial impact on the outcome of overall survival; however, a large heterogeneity was consistently observed.

Eliminating studies that scored 6 on the NOS scale did not alter the unfavorable prognostic effect of Sirt1 overexpression on the overall survival in patients with solid malignancies (*n* = 27, HR: 1.53, 95% CI: [1.20, 1.94], *P* = 0.0006, *I*^2^ = 78%).

### Publication bias

**Figure 5:**
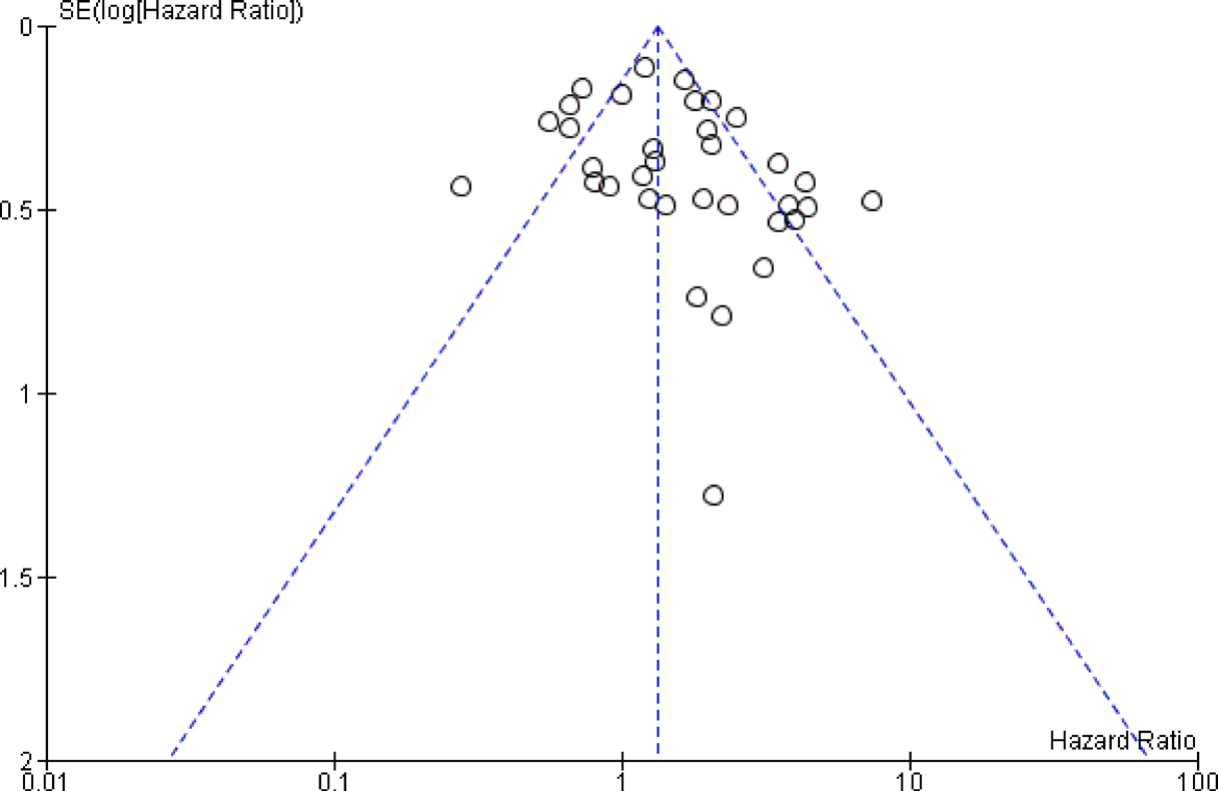
The funnel plots for this meta-analysis

We used funnel plots to visualize publication bias. (Figure 5).

## DISCUSSION

The dual nature of Sirt1 in cancer remains a controversy and could be a consequence of several factors including its different expression levels in various types of carcinoma, its subcellular location, and diverse downstream substrates. Earlier studies predominately reported nuclear Sirt1 expression levels, whereas more recent studies have included cytoplasmic Sirt1 expression levels. It has been proposed that subcellular localization may account for the dual roles of SIRT1 in normal versus cancer cells [47]. One study suggested that the cytoplasmic SIRT1 originates in the nucleus and plays a role in enhancing caspase-dependent apoptosis [48]. All these studies suggested that cytoplasmic expression of Sirt1 is a worse indicator of poor prognosis in cancer patients than the nuclear expression of Sirt1. However, of all the included studies, there were only two articles that discussed the relationship between Sirt1 cytoplasmic expression and patient survival, and regardless, the two studies failed to draw the same conclusion. This inconsistency could be because of different scoring criteria or differing protocols, and therefore, additional studies that precisely illuminate the impact of cytoplasmic Sirt1 expression are needed.

Although an overwhelming number of studies have established evidence that indicates an unfavorable impact of Sirt1 overexpression on patient prognosis in a wide range of carcinomas, several recent investigations revealed a superior survival duration in cases with abnormal expressions of Sirt1. The exact cause for this inconsistency is unknown. Thus, from a clinical perspective, the significance of Sirt1 in patient survival is unknown due to the lack of convincing evidence, and therefore, a comprehensive study is urgently needed.

Based on our knowledge, our study is the first and most versatile meta-analysis that systematically elucidates the prognostic role of Sirt1 overexpression in solid malignancies. Altogether, the results of our analyses strongly support the current mainstream point that Sirt1 redundancy was significantly correlated with patient overall survival in carcinomas. Furthermore, this unfavorable prognostic impact was independent of TNM stages. However, our quantitative analysis found that Sirt1 overexpression was not associated with patient survival in breast cancer, colorectal cancer, and gastric cancer, which was inconsistent with a majority of previous findings, and this contradiction could result from the data collection process. Some of the included study data were acquired from figures in the articles because of a lack of individual patient data. It is also worth mentioning that our subgroup analyses of the correlations between abnormal Sirt1 expression and breast cancer or gastric cancer did not lead to the same conclusions as the meta-analysis [49, 50]. These inconsistencies originated from the differences in literature selection criteria: we excluded data that were not published in English and those from geo database in cases of data duplication.

Apart from the interesting results, there are some limitations to this quantitative meta-analysis. First, the heterogeneity among the studies remained, despite the usage of a random-effects model and subgroup analyses. The heterogeneity could have resulted in outcome bias. Second, we barely explored the correlation between Sirt1 overexpression and patient survival in terms of clinical parameters. Other elements that may contribute to the heterogeneity, such as pathological grade, body mass index, and mean age, were not analyzed due to the lack of sufficient data. Finally, because of a shortage of original individual patient data, we performed a quantitative meta-analysis based mostly on secondary data, which could lead to inaccurate results.

In spite of the limitations mentioned above, there is plenty of pragmatic value in this full-scale, quantitative meta-analysis. First, Sirt1 was identified as a biomarker of overall survival in solid malignancies, especially in liver cancer and lung cancer. Second, we proposed that cytoplasmic rather than nuclear Sirt1 expression deserves more attention. Additional and more in-depth clinical studies are needed because current studies indicate that Sirt1 can serve as a more accurate prognostic predictor in carcinomas.

## MATERIALS AND METHODS

### Search strategy

We performed a thorough electronic search for relevant studies using PubMed and Web of Science that were published before December 2016. The search terms “Sirt1 AND (cancer OR neoplasm OR carcinoma OR malignancy)” were applied, and we initially identified 3474 studies for further examination. Both abstracts and full texts were elaborately screened to exclude irrelevant articles. Additionally, we reviewed the citation lists of the retrieved articles to guarantee the sensitivity of the search process. This procedure was carried out by two authors separately, and discrepancies were resolved by mutual discussions.

### Selection criteria

Studies that met the following criteria were considered eligible and were included in our quantitative meta-analysis: (1) articles written in English and published before Dec. 2016; (2) studies discussing the correlation between Sirt1 expression and patient prognosis in human solid malignancies; (3) a minimal follow-up duration of 3 years; (4) a minimal sample-size of 10 participants; and (5) the diagnosis of solid malignancy was histologically and pathologically confirmed. Studies were excluded on the basis of the following criteria: (1) duplicate or overlapping populations; (2) lack of enough statistical data for further quantification analyses; (3) review articles or case reports; (4) animal studies; and (5) articles based on the Geo database. All evaluations were independently conducted by two authors to ensure the accuracy of the selection process.

### Data extraction

General information, overall survival, cancer-specific survival, disease-free survival, and recurrence-free survival were extracted from qualified studies independently by two investigators. The original survival data for both comparative groups were calculated from the text, tables or Kaplan-Meier curves. The survival information from Kaplan-Meier curves were digitized and extracted using Enguage Digitizer 4.1. Any disagreements were resolved by mutual discussions. All extraction procedures were performed with the aid of predefined standardized extraction forms.

### Methodological quality assessment

Newcastle-Ottawa Scale (NOS) [51] was applied for the quality evaluation of each selected article because all the eligible studies were observational studies. Certain adaptive modifications were made to revise the scale to match the practical needs of the analysis. The scale contained three categories including selection, comparability and outcome, and the maximum score was nine. Methodological high quality studies were those that scored more than six on this scale. The assessment process was conducted independently by two authors.

### Statistical analysis

All quantitative calculations were performed using Review Manager 5.3 (Cochrane Collaboration, Oxford, England). The hazard ratio (HR) at a 95% confidential interval (CI) was used to measure the correlation between Sirt1 expression and patient survival. The data, including the general survival analyses and sub-group comparisons, calculated from the articles were included in the form of generic inverse variables. Heterogeneity among studies was calculated using both the *I*^2^ test and *Q*-test, and *I*^2^ > 25% or *P* < 0.05 was defined as significant heterogeneity; therefore, a random-effects model (the DerSimonian and Laird method) was used. In all other cases, the fixed-effects model (Mantel-Haenszel method) was used. Additionally, we conducted a sensitivity analysis to test the consistency of the selected studies. Publication bias was determined using funnel plots, and *P* < 0.05 signified a statistically significant publication bias. All *P* values were 2-tailed.

## SUPPLEMENTARY MATERIALS FIGURES



## Abbreviations

Sirt1: silent information regulator 1
OS: overall survival
DFS: disease-free survival
RFS: relapse-free survival
CSS: cancer-specific survival
