# Supplementary material for: The prognostic role of Sirt1 expression in solid malignancies: a meta-analysis
Source: Oncotarget. 2017 Jun 15;8(39):66343–51. doi: 10.18632/oncotarget.18494 (PMC5630416; doi:10.18632/oncotarget.18494)
Supplement: Supplementary file 1 [file oncotarget-08-66343-s001.pdf]

The prognostic role of Sirt1 expression in solid malignancies: a meta-analysis

Supplementary Materials

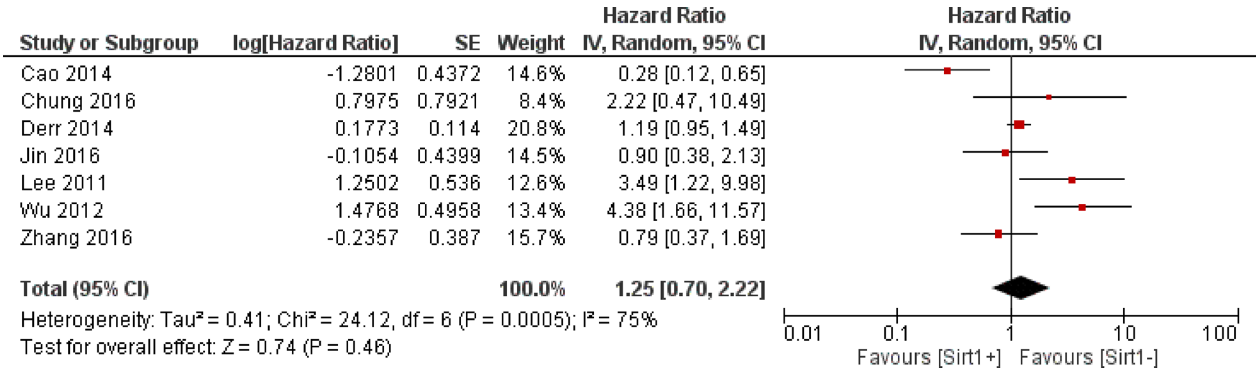

Supplementary Figure 1: The correlation between Sirt1 expression and overall survival of breast cancer.

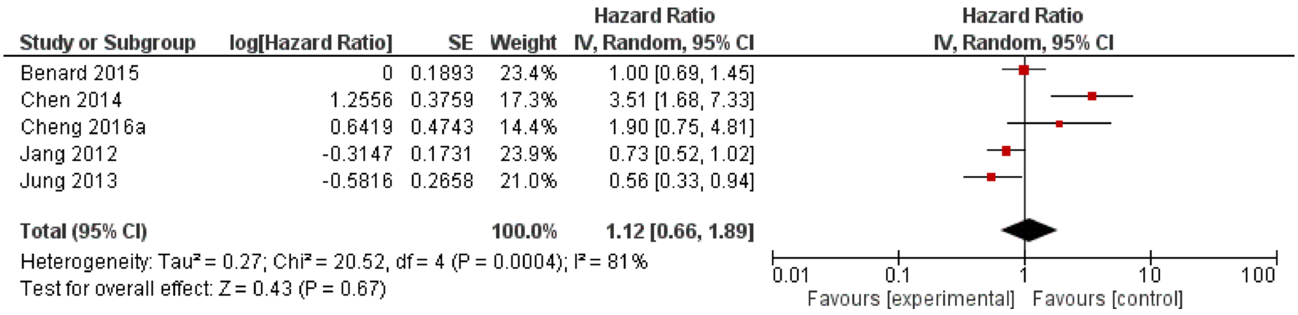

Supplementary Figure 2: The correlation between Sirt1 expression and overall survival of colorectal cancer.

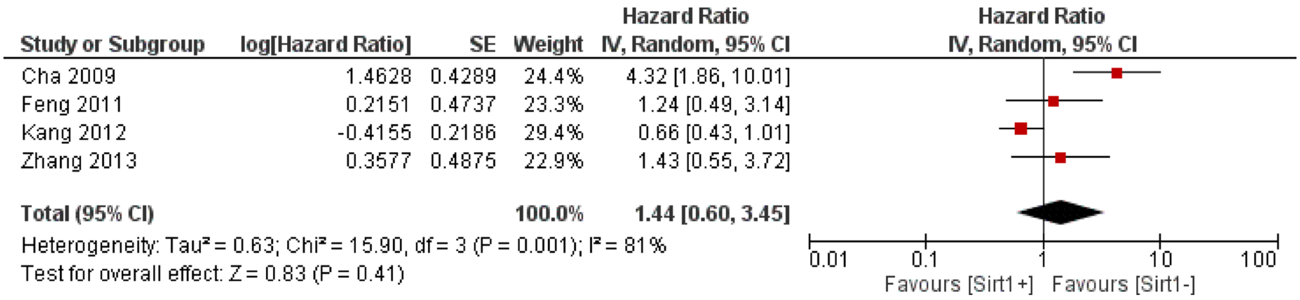

Supplementary Figure 3: The correlation between Sirt1 expression and overall survival of gastric cancer.

Study or Subgroup	log[Hazard Ratio]	SE	Weight	Hazard Ratio IV, Fixed, 95% CI
Benard 2015	0	0.1893	21.8%	1.00 [0.69, 1.45]
Derr 2014	0.1773	0.114	60.2%	1.19 [0.95, 1.49]
He 2016	0.7178	0.327	7.3%	2.05 [1.08, 3.89]
Jin 2016	-0.1054	0.4399	4.0%	0.90 [0.38, 2.13]
Li 2016	0.6043	0.7389	1.4%	1.83 [0.43, 7.79]
Zhang 2016	-0.2357	0.387	5.2%	0.79 [0.37, 1.69]
**Total (95% CI)**			**100.0%**	**1.16 [0.98, 1.38]**

Heterogeneity: Chi² = 5.41, df = 5 (P = 0.37); I² = 8%
Test for overall effect: Z = 1.71 (P = 0.09)

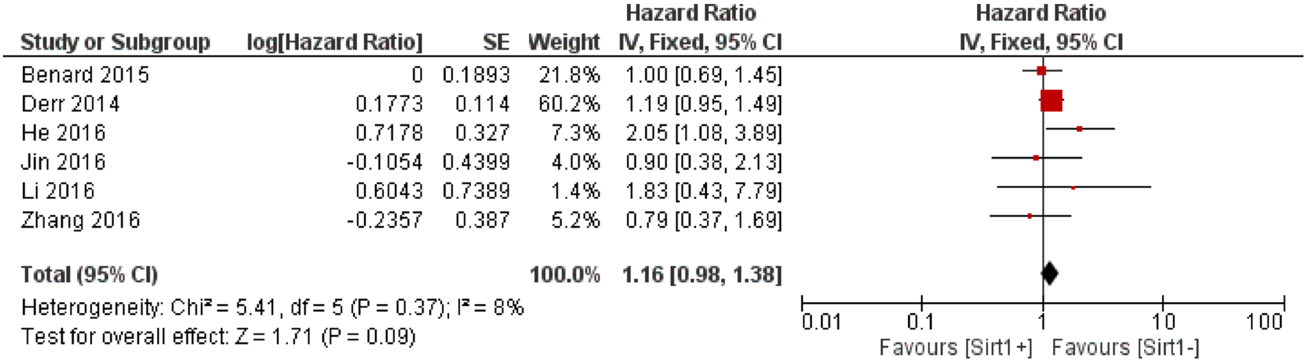

Supplementary Figure 4: The correlation between Sirt1 expression and overall survival of pre-terminal TNM stages.

Study or Subgroup	log[Hazard Ratio]	SE	Weight	Hazard Ratio IV, Random, 95% CI
Cao 2014	-1.2801	0.4372	8.1%	0.28 [0.12, 0.65]
Cha 2009	1.4628	0.4289	8.2%	4.32 [1.86, 10.01]
Cheng 2016a	0.6419	0.4743	7.7%	1.90 [0.75, 4.81]
Hao 2014	0.27	0.3735	8.8%	1.31 [0.63, 2.72]
Jang 2012	-0.3147	0.1731	10.9%	0.73 [0.52, 1.02]
Jang 2012a	0.9235	0.2522	10.1%	2.52 [1.54, 4.13]
Jung 2013	-0.5816	0.2658	10.0%	0.56 [0.33, 0.94]
Kang 2012	-0.4155	0.2186	10.5%	0.66 [0.43, 1.01]
Li 2015	1.3823	0.53	7.1%	3.98 [1.41, 11.26]
Noh 2013	1.3324	0.4916	7.5%	3.79 [1.45, 9.93]
Song 2014	0.5068	0.1488	11.0%	1.66 [1.24, 2.22]
**Total (95% CI)**			**100.0%**	**1.35 [0.87, 2.11]**

Heterogeneity: Tau² = 0.44; Chi² = 69.03, df = 10 (P < 0.00001); I² = 86%
Test for overall effect: Z = 1.33 (P = 0.18)

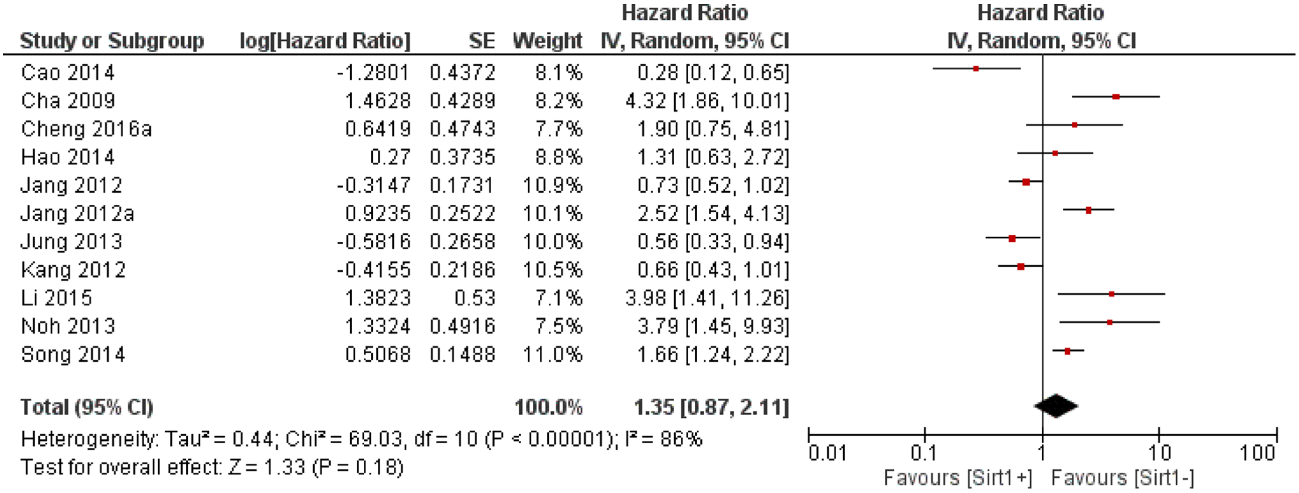

Supplementary Figure 5: The correlation between Sirt1 expression and overall survival of all TNM stages of cancer.

Study or Subgroup	log[Hazard Ratio]	SE	Weight	Hazard Ratio IV, Random, 95% CI
Cha 2009	1.2829	0.3752	14.7%	3.61 [1.73, 7.53]
Derr 2014	0.2807	0.1276	20.6%	1.32 [1.03, 1.70]
Grbesa 2015	0.2546	0.3576	15.1%	1.29 [0.64, 2.60]
Lee 2011	0.9501	0.3709	14.8%	2.59 [1.25, 5.35]
Noguchi 2013	-0.4231	0.1607	20.0%	0.66 [0.48, 0.90]
Noh 2013	0.8033	0.3668	14.9%	2.23 [1.09, 4.58]
**Total (95% CI)**			**100.0%**	**1.58 [0.97, 2.60]**

Heterogeneity: Tau² = 0.29; Chi² = 30.57, df = 5 (P < 0.0001); I² = 84%
Test for overall effect: Z = 1.82 (P = 0.07)

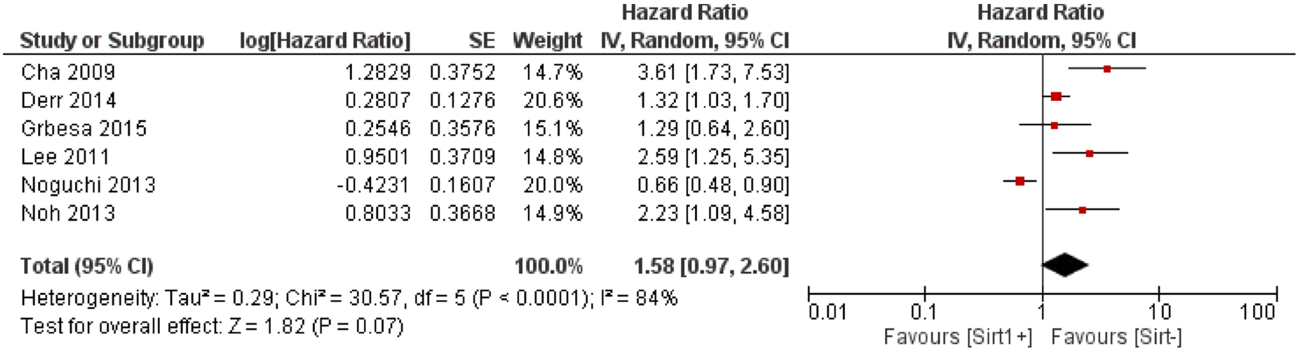

Supplementary Figure 6: The correlation between Sirt1 expression and relapse-free survival.

Study or Subgroup	log[Hazard Ratio]	SE	Weight	Hazard Ratio IV, Random, 95% CI
Cao 2014	-1.2658	0.4317	8.3%	0.28 [0.12, 0.66]
Chen 2014a	0.2469	0.2089	13.8%	1.28 [0.85, 1.93]
Chung 2015	0.1222	0.3674	9.7%	1.13 [0.55, 2.32]
Chung 2016	0.5247	0.2729	12.0%	1.69 [0.99, 2.89]
Jang 2012	-0.1625	0.1369	15.5%	0.85 [0.65, 1.11]
Jang 2012a	0.4266	0.206	13.8%	1.53 [1.02, 2.29]
Jin 2016	0.2852	0.3423	10.3%	1.33 [0.68, 2.60]
Li 2016	0.4253	0.4862	7.3%	1.53 [0.59, 3.97]
Wu 2012	1.2096	0.3818	9.3%	3.35 [1.59, 7.08]
**Total (95% CI)**			**100.0%**	**1.23 [0.88, 1.73]**

Heterogeneity: $Tau^2 = 0.17$; $Chi^2 = 28.05$, df = 8 (P = 0.0005); $I^2 = 71\%$
Test for overall effect: Z = 1.22 (P = 0.22)

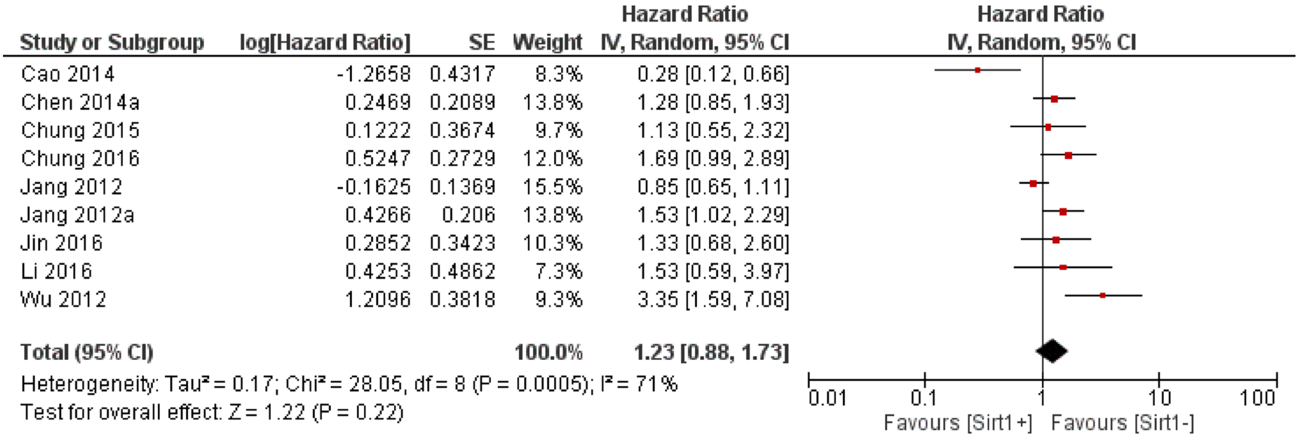

Supplementary Figure 7: The correlation between Sirt1 expression and disease-free survival.

Study or Subgroup	log[Hazard Ratio]	SE	Weight	Hazard Ratio IV, Random, 95% CI
Noguchi 2013	-0.4463	0.1763	38.8%	0.64 [0.45, 0.90]
Noguchi 2014	0.4612	0.1716	38.9%	1.59 [1.13, 2.22]
Noh 2013	1.4855	0.6264	22.3%	4.42 [1.29, 15.08]
**Total (95% CI)**			**100.0%**	**1.40 [0.60, 3.30]**

Heterogeneity: $Tau^2 = 0.46$; $Chi^2 = 18.88$, df = 2 (P $<$ 0.0001); $I^2 = 89\%$
Test for overall effect: Z = 0.78 (P = 0.44)

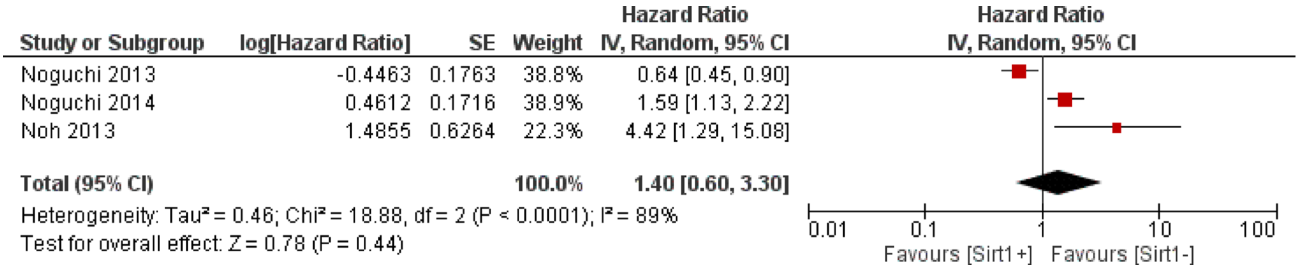

Supplementary Figure 8: The correlation between Sirt1 expression and cancer-specific survival.
